# Genetically-controlled Vesicle-Associated Membrane Protein 1 expression may contribute to Alzheimer’s pathophysiology and susceptibility

**DOI:** 10.1186/s13024-015-0015-x

**Published:** 2015-04-09

**Authors:** Daniel Sevlever, Fanggeng Zou, Li Ma, Sebastian Carrasquillo, Michael G Crump, Oliver J Culley, Talisha A Hunter, Gina D Bisceglio, Linda Younkin, Mariet Allen, Minerva M Carrasquillo, Sigrid B Sando, Jan O Aasly, Dennis W Dickson, Neill R Graff-Radford, Ronald C Petersen, Kevin Morgan for ARUK consortium, Olivia Belbin

**Affiliations:** Department of Neuroscience, Mayo Clinic College of Medicine, Jacksonville, Fl 32224 USA; Human Genetics Division, Cincinnati Children’s Hospital Medical Center, Cincinnati, Ohio 45229 USA; Department of Neurology, St. Olav’s Hospital, Edvard Griegs Gate 8, 7006 Trondheim, Norway; Department of Neuroscience, Norwegian University of Science and Technology, NTNU, 7491 Trondheim, Norway; Department of Neurology, Mayo Clinic College of Medicine, Jacksonville, Fl 32224 USA; Department of Neurology and the Mayo Alzheimer Disease Research Center, Mayo Clinic College of Medicine, Rochester, Minnesota USA; School of Molecular Medical Sciences, Institute of Genetics, Queen’s Medical Centre, University of Nottingham, Nottingham, UK; Memory Disorders Unit, Institute of Biomedical Investigation Sant Pau (IIB Sant Pau), Autonomous University of Barcelona (UAB), Barcelona, Spain

**Keywords:** SNARE, Vesicle-Associated Membrane Protein 1, β-amyloid, Alzheimer’s disease, Synapse

## Abstract

**Background:**

Alzheimer’s disease is a neurodegenerative disorder in which extracellular deposition of β-amyloid (Aβ) oligomers causes synaptic injury resulting in early memory loss, altered homeostasis, accumulation of hyperphosphorylated tau and cell death. Since proteins in the SNAP (Soluble N-ethylmaleimide-sensitive factor Attachment Protein) REceptors (SNARE) complex are essential for neuronal Aβ release at pre-synaptic terminals, we hypothesized that genetically controlled SNARE expression could alter neuronal Aß release at the synapse and hence play an early role in Alzheimer’s pathophysiology.

**Results:**

Here we report 5 polymorphisms in *Vesicle-Associated Membrane Protein 1* (*VAMP1*), a gene encoding a member of the SNARE complex, associated with bidirectionally altered cerebellar *VAMP1* transcript levels (all p < 0.05). At the functional level, we demonstrated that control of VAMP1 expression by heterogeneous knockdown in mice resulted in up to 74% reduction in neuronal Aβ exocytosis (p < 0.001). We performed a case-control association study of the 5 *VAMP1* expression regulating polymorphisms in 4,667 Alzheimer’s disease patients and 6,175 controls to determine their contribution to Alzheimer’s disease risk. We found that polymorphisms associated with increased brain *VAMP1* transcript levels conferred higher risk for Alzheimer’s disease than those associated with lower *VAMP1* transcript levels (p = 0.03). Moreover, we also report a modest protective association for a common *VAMP1* polymorphism with Alzheimer’s disease risk (OR = 0.88, p = 0.03). This polymorphism was associated with decreased *VAMP1* transcript levels (p = 0.02) and was functionally active in a dual luciferase reporter gene assay (p < 0.01).

**Conclusions:**

Genetically regulated *VAMP1* expression in the brain may modify both Alzheimer’s disease risk and may contribute to Alzheimer’s pathophysiology.

**Electronic supplementary material:**

The online version of this article (doi:10.1186/s13024-015-0015-x) contains supplementary material, which is available to authorized users.

## Background

Despite more than 100 years of research on Alzheimer’s disease, the search for drugs that are able to slow or stop disease progression is still ongoing; a search that is further compounded by the fact that if such a disease-modifying drug is to be effective, better understanding of the pre-clinical stage of Alzheimer’s disease is essential. While it is generally accepted that oligomerization of β-amyloid (Aβ) may be the initiating factor in a cascade of neuronal insults and synaptic injury that ultimately lead to neuronal death and early memory loss [1], the mechanisms which precede Aβ oligomerization have yet to be elucidated. One potential mechanism could be an increase in neuronal secretion of Aβ, which in itself would presumably have profound effects on synaptic transmission; Aβ peptides have been shown to bind synapses [2], reduce spine density [3-8] and depress excitatory transmission [9]. The toxic 42 amino acid isoform of Aβ (Aβ42) has been shown to increase the availability and release of synaptic vesicles [10]. Moreover, a feedback loop has been described whereby increased synaptic activity increases Aβ generation and release [11]. It is evident therefore that a correctly functioning secretion system for Aβ is critical for maintaining synaptic homeostatic plasticity and that its malfunction could represent a potential preclinical mechanism that could later trigger Alzheimer pathophysiology.

In 2008, Cirrito et al., reported that an estimated 70% of extracellular Aβ arises from the endocytic-exocytic pathway [11]. Specifically, the full-length amyloid precursor protein (APP) is endocytosed from the plasma membrane where it is sequentially processed by β- and γ-secretases to produce Aβ, which is then secreted from the cell and the APP intracellular domain, which remains localised to the membrane. Fundamental to this process are the SNAP (Soluble N-ethylmaleimide-sensitive factor Attachment Protein) REceptors (SNARE) proteins, which are located on both the vesicular and cytoplasmic membranes [12]. Unification of the SNARE proteins via a common SNARE motif, allows fusion of the Aβ-containing vesicles with the cytoplasmic membrane at pre-synaptic terminals resulting in Aβ release [12]. Moreover, the APP intracellular domain has been demonstrated to directly interact with two SNARE proteins (vesicle-associated membrane proteins; VAMP 1 and 2) within the synaptic vesicles [13], making the VAMP proteins good candidates for functional control of Aβ release. With this in mind, we hypothesized that aberrant SNARE expression may have a direct effect on the levels of extracellular Aβ. We searched for polymorphisms that regulate SNARE expression and found a strong hit for the neuronal SNARE, VAMP1. Here we report an in-depth study of the relationship between *VAMP1* polymorphisms and transcriptional *VAMP1* expression in the brain of Alzheimer’s disease patients and cognitively healthy controls, the correlation between VAMP1 protein expression and neuronal Aβ secretion using primary neurons derived from mice heterogeneously expressing *VAMP1* and a case-control association study of 4,667 Alzheimer’s disease patients and 6,175 controls of Caucasian European descent. Finally, we tested the functional capacity of the *VAMP1* polymorphisms using a dual luciferase reporter gene assay.

## Results

**Figure 1:**
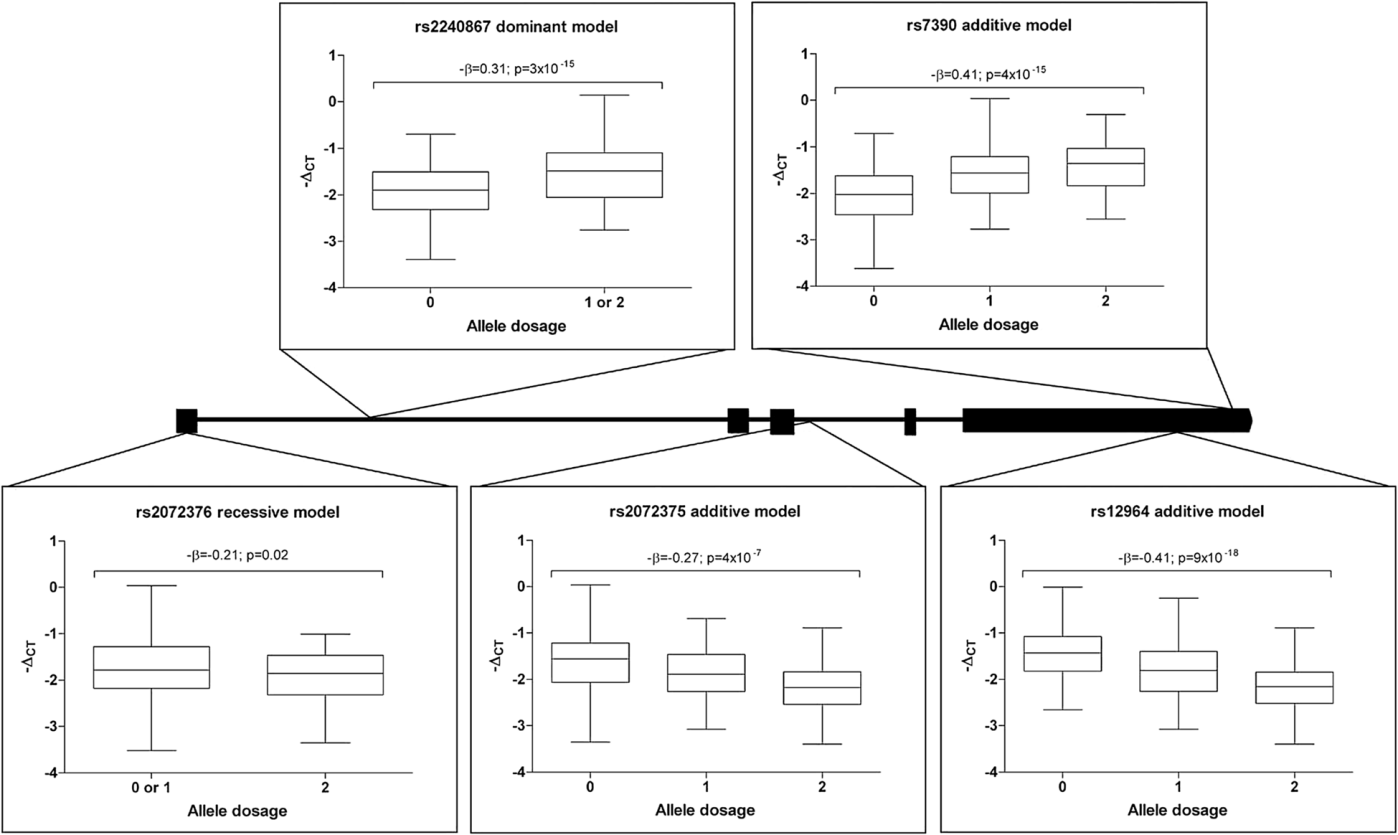
***VAMP1*** **variants are associated with altered** ***VAMP1*** **transcript levels in cerebellum.** Box (25^th^-75^th^ percentiles) and whisker (range of the data) plots are shown for *VAMP1* mRNA expression (-Δ_CT_) in 365 cerebella samples (pooled Alzheimer’s and controls) grouped by *VAMP1* genotype (0, 1, 2 = number of copies of the minor allele). For ease of interpretation, the negative of the Δ_CT_ (-Δ_CT_) are plotted here such that a negative value represents a decrease in expression and a positive value an increase in expression. The β co-efficient (-β for ease of interpretation) and p-values (p) for the logistic regression of Δ_CT_ versus *VAMP1* genotype (adjusted for age, sex and *APOE* ε4 allele) are given. Analyses were performed following additive, dominant and recessive models; the best model (lowest p-value) for each polymorphism is shown. For results in AD and Control subsets and for all models tested, see Additional file 1: Table S3. The location of each polymorphism within VAMP1 is indicated. The scaled schematic represents the full *VAMP1* sequence (line) in the 5′ to 3′ direction including exons (boxes) and 3′untranlsated region (arrowed box).

In order to determine whether SNARE expression was under the transcriptional control of genetic variants, we searched for single nucleotide polymorphisms associated with altered SNARE mRNA transcript expression using a publically available database [14]. The platform included genotypes for 408,273 polymorphisms and measurements of 54,675 transcripts in Epstein-Barr virus-transformed lymphoblastoid cell lines. Our search, which focused on SNAREs that are robustly expressed in the brain (APBA1, SNAP25, STX1A, STXP1, VAMP1, VAMP2), revealed a strong hit for *VAMP1*. All 8 polymorphisms included on the platform within *VAMP1* and the *VAMP1* 3′ untranslated region showed unequivocal association with altered *VAMP1* expression (all p < 3.7×10^-4^; Additional file 1: Table S1). In order to confirm the association in a more relevant tissue, we measured *VAMP1* mRNA and genotyped the *VAMP1* region in 365 post-mortem cerebellum samples (192 AD, 173 controls, Table 1A. For genotype counts see Additional file 1: Table S2A). To ensure that we had good coverage of *VAMP1*, we identified 5 linkage disequilibrium (LD) blocks within the *VAMP1* locus (Additional file 2: Figure S1) using genotype data from the Caucasian European (CEU) population published by the HapMap project (www.hapmap.org). One polymorphism from each LD block was genotyped (Additional file 2: Figure S1) giving 80% coverage of all genotyped polymorphisms at a minor allele frequency (MAF) >1%. We tested for association of genotypes at these 5 polymorphisms with cerebellar *VAMP1* transcript levels adjusting for age, sex and the *APOE* ε4 allele using dominant, additive and recessive models (For full data see Additional file 1: Table S3). The *VAMP1* transcript levels grouped by *VAMP1* genotype are plotted in Figure 1. All 5 polymorphisms were associated with altered *VAMP1* transcripts. The strongest associations were observed for the two most 3′ variants; rs7390 with increased *VAMP1* expression (-β coefficient = 0.41, p = 4×10^-15^) and rs12964 (-β coefficient = -0.41, p < 2×10^-9^) with decreased expression (for ease of interpretation, the negative value of the β coefficients are reported here such that a negative value represents a decrease in expression and a positive value an increase in expression). These associations that are equivalent in direction and effect size to those reported by Dixon et al. in lymphoblastoid cells; rs7390 -β = 0.51, p = 1×10^-9^ (same allele tested) and rs12964 -β = 0.3, p = 3×10^-5^ (opposing allele tested), indicate that the genetic control of *VAMP1* expression is independent of tissue type. The association of rs7390, rs12964, rs2072376 and rs2240867 were also confirmed in both the Alzheimer’s disease (all p < 8.4×10^-5^) and control (all p < 2.9×10^-3^) subsets (Additional file 1: Table S3), indicating that the transcriptional regulation is also independent of diagnosis. The exception was rs2072376, which was associated with altered expression in the controls (p = 0.003) but not Alzheimer’s disease patients (p = 0.8). Expression levels of *VAMP1* did not differ between diagnosis groups (p = 0.41). Overall, these data suggest that *VAMP1* transcription across tissues may be controlled by polymorphisms located at several locations within *VAMP1* and that, in the case of rs2072376, this regulation may be disrupted in the Alzheimer’s disease subgroup.

**Table 1:** **Summary of the patient samples included in this study**

	**Total**	**CTRLs**				**AD**			
**Series**	**N**	**N**	**%F**	**%ε4+**	**Age**	**N**	**%F**	**%ε4+**	**Age**
**A)** Mayo postmortem	365	173	35.3	26.0	71.7	192	51.6	63.0	73.5
**B)** All	10,842	6,175	54.4	23.4	77.6	4,667	61.1	62.3	76.6
Mayo Clinic	6,307	4,250	54.0	23.3	78.7	2,057	60.7	59.9	79.3
NCRAD	910	209	61.7	16.3	78.3	701	64.8	78.5	75.2
Norway	927	569	59.6	24.6	74.9	358	69.8	63.1	79.4
ARUK	2,698	1,147	51.6	24.2	74.7	1,551	58.0	58.0	73.0

Demographic details are shown for **(A)** Samples taken from the cerebellum of autopsy-confirmed AD patients and controls and used for genotype versus mRNA analyses and **(B)** Samples from the Mayo Clinic, Indiana, Norway and Alzheimer’s Research UK (ARUK) Consortium case-control series used for the case-control association study. N; number of samples, %F; percent females, %ε4; percent *APOE ε4* carriers, Age; years.

**Figure 2:**
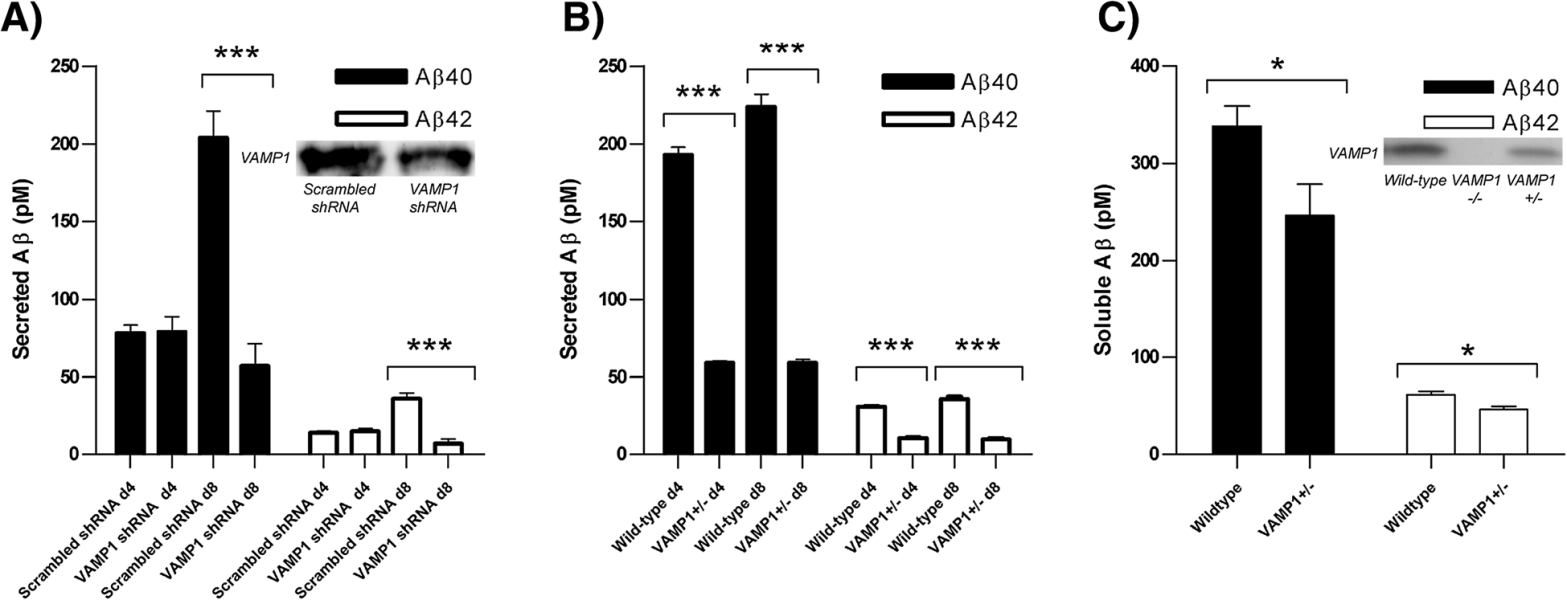
**Lowering** ***VAMP1*** **transcript and VAMP1 protein expression in primary neurons reduces Aβ secretion.** Levels of Aβ40 and Aβ42 species secreted into the media were measured in **A)** mouse primary neurons subjected to shRNA-mediated knockdown of *VAMP1* (*VAMP1* shRNA) versus non-target neurons treated with scrambled shRNA (n = 7), and **B)** mouse primary neurons of transgenic mice with heterogeneous knockdown of VAMP1 (VAMP1+/-) versus Wild-type mice (n = 6). Media were tested for Aβ at day 4 (d4) and day 8 (d8) of neuronal culture. **C)** Soluble Aβ40 and Aβ42 were also measured in brain extracts from Wild-type and VAMP1+/- mice. Bars represent mean values across replicates. Error bars represent standard error of the mean. *p > 0.05, ***p < 0.0001 for t-test. Representative Western blot images showing reduced VAMP1 expression levels in the VAMP1 shRNA treated versus non-target neurons **(A)** and in brains of VAMP1+/- and VAMP1-/- versus wild-type mice **(C)** are shown.

To test our hypothesis that altered *VAMP1* expression in the brain could affect neuronally secreted Aβ levels, we performed shRNA-mediated knockdown of *VAMP1* in primary mouse neurons, achieving a 37.6% reduction in VAMP1 protein expression (Figure 2A). The levels of the two most abundant Aβ species (Aβ1-40 and Aβ1-42) secreted into the cell media were measured by ELISA after 4 and 8 days of culture (Figure 2A). After 4 days, we found that secreted Aβ40 and Aβ42 levels were low in both the non-target neurons treated with scrambled shRNA and *VAMP1* shRNA-treated neurons. However, by 8 days culture we observed an increase in Aβ40 and Aβ42 in the non-target but not in the *VAMP1* shRNA-treated neurons; *VAMP1* shRNA-treated neurons secreted 72% less Aβ40 (p < 0.0001) and 81% less Aβ42 (p < 0.0001) than non-target neurons (n = 7). Compared to other Aβ species, Aβ42 has increased aggregation properties and is believed to be largely responsible for the toxic fibrillar aggregates found in the Alzheimer’s disease brain. Consequently, an increased ratio of Aβ42/40 species in the brain can be a good indicator of underlying Alzheimer’s disease pathology. Notably, we found a decreased Aβ42/40 ratio in shRNA-treated versus non-target neurons (0.12 vs 0.17, respectively). To confirm that this correlation between reduced VAMP1 protein expression and Aβ secretion was not an indirect effect of the shRNA knockdown on the functional capacity of the neurons, we next sought to confirm these findings in primary neuronal cultures from mice heterogeneously expressing *VAMP1*. The *VAMP1+/-* mice were found to express 56% less VAMP1 protein than wildtype mice (Figure 2B). After 4 days culture (Figure 2B), we found a 70% reduction in Aβ40 (p < 0.0001) and 65% reduction in Aβ42 (p < 0.0001) secreted into the media of VAMP1+/- versus wt neurons (n = 6). Moreover, similar reductions were also observed after 8 days of culture (Aβ40 = 74%, p < 0.0001, Aβ42 = 73%, p < 0.0001). However, unlike in the previous culture, we found no change in the Aβ42/40 ratio in neurons of VAMP1+/- mice compared to those from wt mice (0.16 versus 0.16, respectively at day 8). These findings in two primary neuronal cultures support our hypothesis that a decrease in VAMP1 protein expression is directly associated with a decrease in the total levels of Aβ (the pathological protein found in the Alzheimer’s disease brain) exocytosed from neurons. Moreover, VAMP1 +/- mice had a reduced pool of soluble Aβ40 and Aβ42 in the brain of 10-day old VAMP1+/- compared with wildtype mice (Figure 2C), albeit that is impossible to determine from these brain extracts whether this reduction in Aβ is due to a reduction in Aβ secretion or Aβ production.We next sought to determine whether decreased VAMP1 expression could have a protective role against developing Alzheimer’s disease.

**Figure 3:**
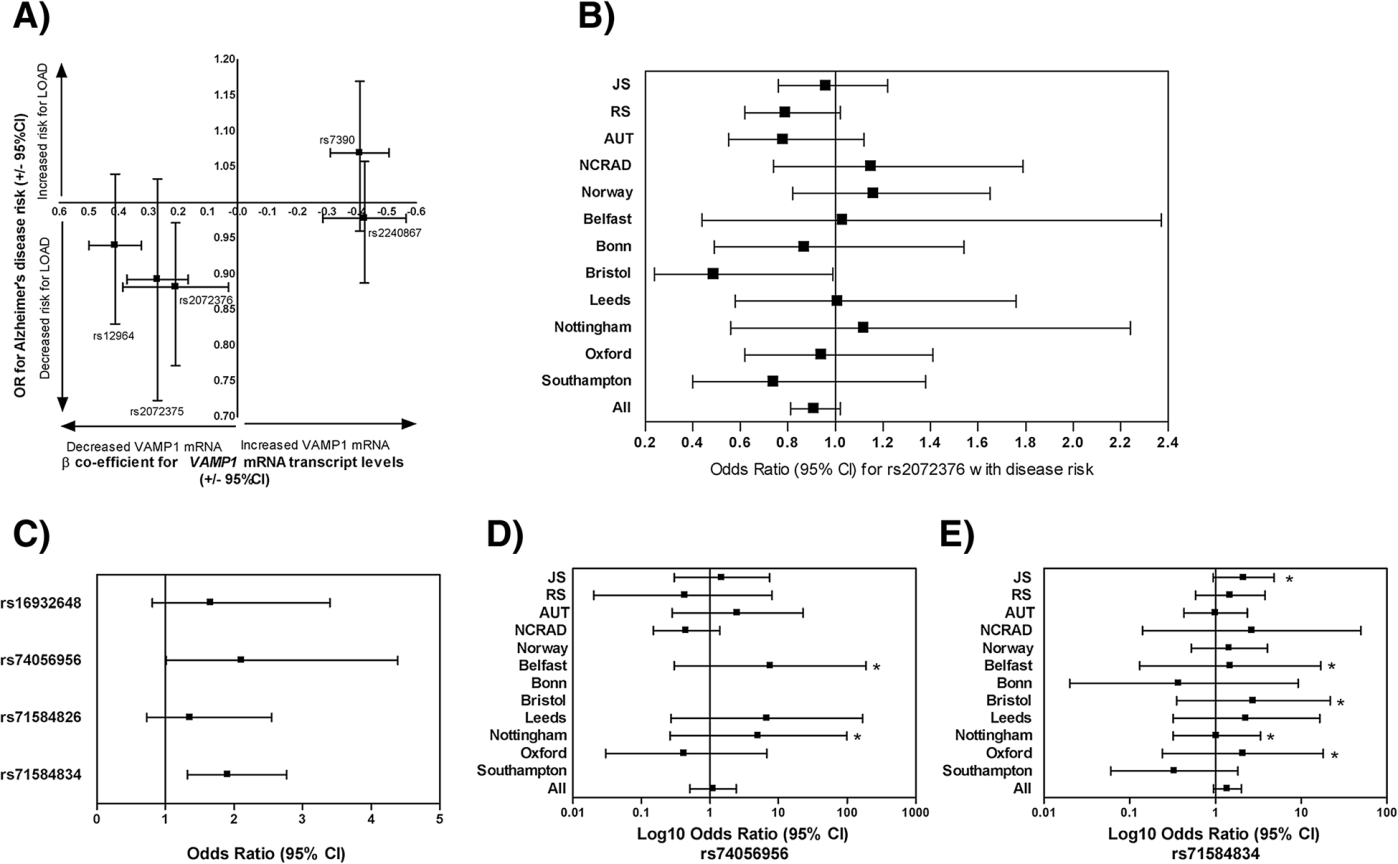
***VAMP1*** **polymorphisms with increased** ***VAMP1*** **brain expression confer higher risk for Alzheimer’s disease. (A)** The odds ratios and 95% confidence intervals (95% CI) were calculated by binary logistic regression using genotype of 5 common *VAMP1* polymorphisms, age, sex and *APOE* ε4 allele as predictive variables for diagnosis were performed following additive, dominant and recessive models; the best model (lowest p-value) for each polymorphism is shown plotted on the y-axis. A significant protective effect of rs2072376 can be seen (OR = 0.88, p = 0.03). The β-coefficients (+/- standard error of the mean) for the same polymorphisms with cerebellar *VAMP1* mRNA expression are plotted on the x-axis. The polymorphisms associated with increased *VAMP1* brain expression (rs7390 and rs2240867) have higher ORs for Alzheimer’s disease susceptibility than those associated with decreased expression. **(B)** Meta analyses across subpopulations (Jacksonville; JS, Rochester; RS, Autopsy-confirmed; AUT) for the rs2072376 polymorphism shows different effect sizes across each population. The population meta-analysis for all samples was not significant (p = 0.62). **(C)** The odds ratios and 95% confidence intervals (95% CI) were calculated for 4 rare *VAMP1* polymorphisms (adjusted for age, sex and *APOE* ε4 allele). A significant association was observed for rs74056956 and rs71548434. Meta analyses across subpopulations for rs74056956 **(D)** and **(E)** rs71548434 show different effect sizes across each population and were not significant (p = 0.80 and 0.10, respectively). The log10 of the odds ratio and 95% CI are plotted on the x-axis for better visualization. The populations in which the genotypes deviated from Hardy Weinberg equilibrium are marked by an asterisk.

In order to determine whether variants that control *VAMP1* expression are associated with altered susceptibility to late-onset Alzheimer’s disease, we genotyped the 5 *VAMP1* polymorphisms in our large case-control series (Table 1B) of Caucasian European descent from the Mayo Clinic, National Cell Repository for Alzheimer’s disease (NCRAD) and Norway (n = 8,144) and utilized the genotypes available for 2 of the polymorphisms from the Alzheimer’s Research United Kingdom (ARUK) consortium case-control series (n = 2,698). Logistic regression (adjusting for age, sex and *APOE* ε4 allele) was performed for dominant, additive and recessive models (for full data see Additional file 1: Table S4). Interestingly, as shown in (Figure 3), the odds ratios (OR) for the polymorphisms associated with increased cerebellar *VAMP1* expression (rs7390; OR = 1.07 and rs2240867; OR = 0.98, mean OR = 1.025) were significantly higher (p = 0.03) i.e. more carrier greater risk for Alzheimer’s disease than the polymorphisms associated with decreased *VAMP1* expression (rs12964; OR = 0.94, rs2072375; OR = 0.89, rs2072376; OR = 0.88, mean OR = 0.90). However, while significant associations for variants rs7390, rs2072375 and rs2072376 (p < 0.05) with altered risk were observed in several of the subpopulations (Additional file 1: Table S4), only the association of rs2072376 remained (p = 0.03) when analyzing the total dataset (6,175 controls, 4,667 AD patients) and only when assuming a recessive model (OR = 0.88, p = 0.03). As shown in Figure 3B, despite the low heterogeneity of these case-control series (0%, p = 0.62), the different effect sizes across the subpopulations meant that the association of rs2072376 did not survive meta-analysis across each series (OR = 0.91, p = 0.11). Overall, these data are, at best, suggestive of a common polymorphism, rs2072376 (MAF = 41%), in *VAMP1* that is associated with decreased cerebellar *VAMP1* expression that may have a modest protective effect against Alzheimer’s disease.

We next sought to determine whether rare variants in the region could be associated with Alzheimer’s disease susceptibility. Sequencing the 28,440 base pair region containing *VAMP1* (+/-20 kb) in 300 Alzheimer’s disease cases and 300 controls (95% power to detect all variants with MAF > 1%), we identified 10 variants, 5 of which were subsequently genotyped in the remaining case-control series (10,842 samples). Of these 5 variants (all MAF < 0.3%), rs77069473 was discarded due to a minor allele homozygote frequency higher than that of the heterozygotes (Additional file 1: Table S5). Of the remaining 4 variants, rs74056956 (OR = 2.11, p = 0.05) and rs71584834 (OR = 1.91, p = 0.0006) were associated with increased Alzheimer’s disease risk (Figure 3C). However, it must be noted that the genotypes deviated from Hardy-Weinberg equilibrium in several of the subpopulations for both of these variants (marked by asterisk in Figure 3D for rs74056956 and Figure 3E and in Additional file 1: Table S5) and neither association remained following meta-analyses across the subpopulations. Unfortunately, due to their low frequency, to determine whether, like their common counterparts, these rare *VAMP1* variants are associated with altered *VAMP1* expression would require a much larger collection of postmortem samples than is currently available.

**Figure 4:**
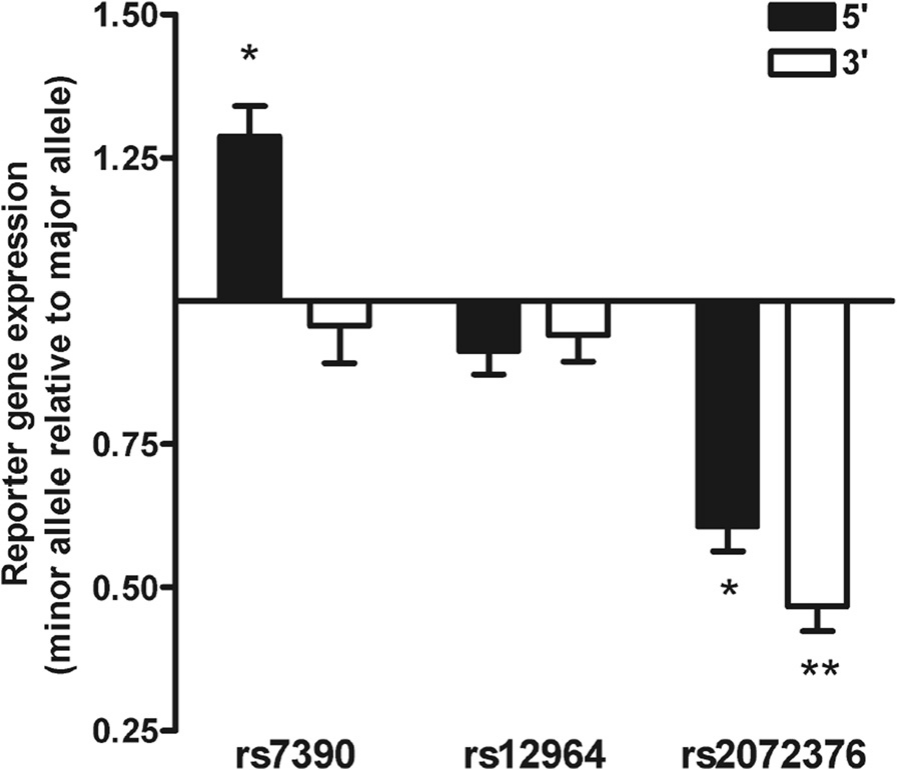
**A** ***VAMP1*** **variant associated with decreased cerebellar** ***VAMP1*** **is a functionally active repressor of expression.** The reporter gene expression (ratio of firefly:renilla) for the minor allele sequence relative to that of the major allele sequence are plotted for the rs7390, rs12964 and rs2072376 variants transfected in HepG2 cells. DNA sequences were cloned 5′ (filled boxes) and 3′ (clear boxes) to the promoter. Unpaired T-tests were used to test for altered reporter gene expression between major and minor sequences. Error bars represent SEM (standard error of the mean). *p < 0.05, **p < 0.01 for t-test.

Finally, in order to determine the functionality of the *VAMP1* polymorphisms*,* we employed a dual luciferase reporter gene assay to test the best two expression-associated polymorphisms (rs7390 and rs12964) and the best Alzheimer’s disease susceptibility polymorphism (rs2072376) in a human hepatocellular carcinoma line (HepG2). When comparing the activity of the minor vs major allelic sequence of the 3 polymorphisms cloned 5′ to the promoter (Figure 4; black bars), changes in reporter gene expression were observed in directions consistent with those we report with cerebellar *VAMP1* expression for rs7390 (1.3-fold increase; p = 0.01) and rs2072376 (0.6-fold decrease; p = 0.01). For rs12964, only a trend towards a 0.9-fold decrease (p = 0.06) was observed. When the sequences were cloned 3′ to the promoter (white boxes), the association of rs2072376 with decreased reporter gene expression remained (0.5-fold decrease; p = 0.007), suggesting that the functional capacity of the rs2072376 sequence is independent of its relative location to the promoter and is therefore a strongly suggestive that this is a true functional variant. These findings demonstrate that the rs2072376 variant, for which we report an association with decreased cerebellar *VAMP1* expression and a protective association with reduced Alzheimer’s disease susceptibility, has functional repressor activity.

## Discussion

Here we have identified a strong association of common *VAMP1* polymorphisms with *VAMP1* cerebellar transcript levels in Alzheimer’s disease and control brains, with the strongest correlation at the 3′ end of the gene with increased *VAMP1* expression (rs7390 and rs12964, all p < 2.4×10^-7^). To put this in a functional context, we demonstrated in neuronal cultures that decreased VAMP1 protein expression by shRNA knockdown of *VAMP1* is associated with up to 74% decreased Aβ40 and up to 73% decreased Aβ42 secretion (p < 0.0001). Notably, we did not find a significant reduction in Aβ secretion in VAMP1 -/- compared with wild-type mice (data not shown). This could indicate that there is a compensatory mechanism for neuronal secretion in mice when VAMP1 is completely ablated, which is not apparent in VAMP1+/- mice.

The reduction in extracellular Aβ levels in VAMP1 +/- neurons is likely due to the role of VAMP1 as part of the SNARE complex, which is responsible for mediating the fusion of Aβ-containing vesicles with the pre-synaptic membrane, resulting in Aβ exocytosis. We propose that as a consequence of reduced transcriptional expression, reduced neuronal VAMP1 protein levels would impede SNARE complex formation and in turn lead to reduced recycling of Aβ to the membrane for exocytosis., However, we cannot rule out other mechanisms by which VAMP1 expression may affect extracellular Aβ levels (e.g. altered Aβ production, degradation or reuptake).

Since deposition and oligomerization Aβ is a key pathological hallmark of the Alzheimer’s brain, our data led us to propose that genetic variation at the *VAMP1* locus may be associated with altered susceptibility against Alzheimer’s disease. Our large case-control association study of 5 independent *VAMP1* polymorphisms in 4,667 Alzheimer’s disease patients and 6,175 controls revealed that the odds ratio for Alzheimer’s disease susceptibility was significantly higher for *VAMP1* polymorphisms associated with increased *VAMP1* transcript expression than for those with decreased *VAMP1* transcript expression (p = 0.03). Moreover, we report a modest association of a common polymorphism, rs2072376 (MAF = 0.40), located at the 5′ end of *VAMP1*, with decreased risk for Alzheimer’s disease (OR = 0.88, p = 0.03). This same polymorphism was associated with decreased cerebellar *VAMP1* transcript levels (-β = -0.21; p = 0.02) and demonstrated functional repressor activity *in vitro* (p < 0.01), thus supporting our hypothesis that decreased *VAMP1* expression may be protective against Alzheimer’s disease.

This study has primarily focused on the specific role of VAMP1 in Aβ secretion and its association with Alzheimer’s disease pathophysiology and susceptibility. However, it must be noted that haploinsufficiency of VAMP1 has been reported to cause dominant hereditary spastic ataxia [15] and that schizophrenic patients have lower VAMP1 levels in the superior temporal gyrus than unaffected controls [16]. Furthermore, a null mutation in *VAMP1,* which arose spontaneously in C3H/HeSnJ mice, was associated with muscle wastage, neurological defects and eventual death [17]. Therefore, while there is a precedent for abnormal VAMP1 expression with several unrelated neurological disorders, this is the study is the first to report a potentially protective effect of VAMP1. This leads to the possibility that on the one hand, reduced VAMP1 expression can lead to a global dysfunction in neuronal transmission, which in turn may lead to muscle wastage, ataxia or schizophrenia, while on the other hand, when resulting in a specific reduction in neuronal Aβ secretion, may be protective against Alzheimer’s disease. It would therefore be interesting, but beyond the scope of this study, to determine whether individuals with reduced cerebral VAMP1 expression have other neurological conditions.

One proviso of this study is that the protective association of rs2072376 with Alzheimer’s disease susceptibility, as assessed by logistic regression, was modest (p = 0.03) and did not hold following meta-analyses across each subpopulation (p = 0.11). Although it must be noted that unlike the regression, the meta-analyses cannot take into account other variables (age, sex and *APOE* ε4 allele), the meta-analyses do indicate a population-specific effect size. These findings highlight the importance of confirming this association in further independent case-control series before a conclusive association between *VAMP1* genotype and Alzheimer’s disease susceptibility can be claimed. Similarly, we report 2 rare variants (MAF < 0.005) that confer risk for Alzheimer’s disease risk (rs74056956; OR = 0.91 p = 0.05 and rs71584834; OR = 2.11, p = 0.0006) that should be confirmed in further independent case-control studies.

## Conclusions

These data suggest that while the contribution of *VAMP1* genotype to Alzheimer’s disease risk is at best modest, what is clear is that control of the expression of this particular SNARE can affect a key cellular process in the pathophysiology of Alzheimer’s disease by altering the neuronal secretion of the Aβ peptide. These data point towards dysfunctional synaptic recycling of the Aβ peptide as an early pathological mechanism that could trigger a series of Aβ-related neuronal insults eventually leading to Alzheimer’s disease. Consequently, we propose that genetic variants in genes encoding other SNARE proteins may also be modifiers of Alzheimer’s pathology and/or susceptibility.

## Methods

### Ethics statement

Approval was obtained from the ethics committee or review board of each institution responsible for the ascertainment and collection of samples (Mayo Clinic College of Medicine, Jacksonville, FL and Mayo Clinic College of Medicine, Rochester, MN, USA, National Cell Repository for Alzheimer’s disease, Indianapolis. IN, USA, Department of Neurology, St. Olav’s Hospital, Norway, Department of Neuroscience, Norwegian University of Science and Technology, Norway, ^7^School of Molecular Medical Sciences, Institute of Genetics, Queen’s Medical Centre, University of Nottingham, Nottingham and all institutions in the ARUK consortium). Written informed consent was obtained for all individuals that participated in this study.

### USA case-control subjects

The case-control series consisted of Caucasian subjects of European descent from the United States ascertained at the Mayo Clinic (2,057 late-onset Alzheimer’s disease, 4,250 controls) or through the National Cell Repository for Alzheimer’s Disease (NCRAD: 701 late-onset Alzheimer’s disease, 209 control). All subjects ascertained at the Mayo Clinic in Jacksonville, Florida (JS: 868 LOAD, 1,472 controls) and at the Mayo Clinic in Rochester, Minnesota (RS: 600 late-onset Alzheimer’s disease, 2,408 control) were diagnosed by a Mayo Clinic neurologist. The neurologist confirmed a Clinical Dementia Rating score of 0 for all JS and RS subjects enrolled as controls; cases had diagnoses of possible or probable late-onset Alzheimer’s disease made according to NINCDS-ADRDA criteria [18]. In the autopsy-confirmed series (AUT: 589 late-onset Alzheimer’s disease, 370 control) all brains were evaluated by Dr. Dennis Dickson and came from the brain bank maintained at the Mayo Clinic in Jacksonville. The diagnosis of confirmed Alzheimer’s disease was made according to NINCDS-ADRDA criteria. All late-onset Alzheimer’s disease brains analyzed in the study had a Braak score of 4.0 or greater. Brains employed as controls had a Braak score of 2.5 or lower but often had brain pathology unrelated to AD and pathological diagnoses that included vascular dementia, fronto-temporal dementia, dementia with Lewy bodies, corticobasal degeneration, argyrophilic grain disease, multi-system atrophy, amyotrophic lateral sclerosis, and progressive supra-nuclear palsy. No subjects in this study carried familial Alzheimer’s disease mutations in APP or Presenilin genes. The frequency of *APOE* ε4+ individuals, females and mean age at diagnosis/entry in the late-onset Alzheimer’s disease cases and controls for each series are shown in Table 1 and Additional file 1: Table S2.

### Norway case-control subjects

Samples from Norway consisted of 358 patients diagnosed with probable or possible AD and 569 cognitively-normal controls, all ethnic Norwegians. The patients were neurological and geriatric patients recruited from the University Hospital of Trondheim, the district hospital in Namsos and patients from nursing homes in central Norway. Further details regarding thee samples can be found in previous publications [19,20].

### ARUK case-control subjects

Samples from a total of 2,698 subjects were obtained from seven Alzheimer’s Research UK (ARUK) network centers (Queen’s University Belfast, University of Bristol, University of Leeds, University of Manchester, University of Nottingham, the Oxford Project to Investigate Memory and Ageing (OPTIMA) and University of Southampton) and the University of Bonn, Germany. All samples were from subjects who were diagnosed clinically using NINCDS-ADRDA criteria [18]. All patients with evidence of an autosomal dominant Alzheimer’s disease trait, or where a first degree relative had been diagnosed with familial Alzheimer’s disease, were excluded. Since there were no controls available for the Manchester series, the Manchester Alzheimer’s disease samples were combined with those in the Oxford series when the individual series were analyzed. The frequency of *APOE* ε4+ individuals, females and mean age at diagnosis/entry in the late-onset Alzheimer’s disease cases and controls for each series are shown in Table 1 and Additional file 1: Table S2.

### DNA isolation

For the Mayo Clinic samples, DNA was isolated from whole blood using an AutoGen instrument (AutoGen, Inc, Holliston, MA). The DNA from AUT samples was extracted from cerebellum using WizardH Genomic DNA Purification Kits (Promega Corp., Madison, WI). DNA from the Mayo Clinic Rochester and the autopsy-confirmed series was scarce, so samples from these two series were subjected to whole genome amplification using the Illustra GenomiPhi V2 DNA Amplification Kit (GE Healthcare Bio-Sciences Corp., Piscataway, NJ). For the ARUK samples, genomic DNA was extracted from whole blood samples or brain tissue using the QIAamp DNA blood mini kit (Qiagen, Crawley, West Sussex, UK).

### Genotyping of variants

All genotyping was performed at the Mayo Clinic in Jacksonville using TaqManH SNP Genotyping Assays in an ABI PRISMH 7900HT Sequence Detection System with 384-Well Block Module from Applied Biosystems, California, USA. The genotype data was analyzed using the SDS software version 2.2.2 (Applied Biosystems, California, USA). Fifteen percent of the samples assayed were of known genotype, determined by sequencing and 10% were genotyped in duplicate as a quality assurance measure. The data were only accepted when there was 100% concordance between duplicate samples. Details of the variants investigated in this study, including location, allele frequencies, Hardy-Weinberg equilibrium p-values and genotype counts can be found in Additional file 1: Tables S2 and S5.

### Sequencing of *VAMP1*

In each of the 300 subjects screened, we evaluated amplicons that contained a total of 28,440 bp. This DNA included all exons, introns, and 20 kb of 5′ and 3′ flanking sequence. PCR primer pairs were designed to screen the targeted conserved segments via denaturing high performance liquid chromatography (dHPLC). PCR amplicons were generated with 20 ng of DNA in a 50 ul PCR containing 0.2 mM forward primer, 0.2 mM reverse primer, 200 mM dNTPs, 5 ul of 106 reaction buffer with 25 mM MgSO (Transgenomic, Inc.), and 1 Unit of OptimaseH Polymerase (Transgenomic, Inc.), using one of the following three conditions in a Hybaid thermocycler: 60–50 Touchdown, 62–57 Touchdown, or 55–45 Touchdown. Each PCR product was denatured at 95 uC for 10 min and cooled slowly to 25 uC at a rate of 0.03 uC/sec to encourage heteroduplex formation. 5 ml of each sample was injected into a DNASepH HT Cartridge 6.5 mm 637 mm (Transgenomic, Inc.) and analyzed in a WAVE DHPLC instrument (Transgenomic, Inc.) to identify heterozygotes. The optimal oven temperature and WAVE OptimizedH buffer gradient for DHPLC analysis of each amplicon was selected using the Navigator TM4 software (Transgenomic, Inc.). Samples were categorized as either heteroduplexes or homoduplexes, based on the resulting elution profiles as recommended by Transgenomic, Inc. Representative homoduplexes and heteroduplexes from each amplicon were sequenced in order to determine the nature of the DNA variation underlying each heteroduplex profile. 20 ml of remaining PCR product from the selected samples were purified for the sequencing reaction using the MultiScreenH PCR96 Filter Plates (Millipore). Sequencing in the forward and reverse orientation was performed at the Molecular Biology Core Facility at the Mayo Clinic, Rochester, MN as described on their website (http://www.mayo.edu/research/core-resources/molecular-biology-core/services).

### Measurement of *VAMP1* mRNA Expression

Total RNA was extracted from 365 samples of cerebellum from late-onset Alzheimer’s disease brains and controls (a subset of the Autopsy-confirmed samples from Mayo Clinic) using an ABI PRISM 6100 Nucleic Acid PrepStation and the Total RNA Isolation Chemistry kit from Applied Biosystems. RNA was reverse transcribed to single-stranded cDNA using the High-Capacity cDNA Archive Kit from Applied Biosystems. Realtime quantitative PCR was performed in triplicate for each sample using ABI TaqMan Low Density expression Arrays (384-Well Micro Fluidic Cards) with a pre-validated TaqMan Gene Expression Assay. 18 s ribosomal RNA (18 s rRNA) was used as the endogenous control for the relative quantification of *VAMP1* mRNA. Real-time PCR cycle threshold (C_T_) raw data was collected and exported using the ABI PRISMH SDS software version 2.2. The variable C_T_ within the raw data file indicates the PCR cycle number at which the amount of amplified gene target reaches a fixed threshold. The variable ΔC_T_ denotes the difference between the averaged C_T_ values for the *VAMP1* transcript and that for the reference 18S rRNA transcript. The ΔC_T_ values calculated from each sample were used as quantitative phenotypes to determine associations between *VAMP1* genotypes and the level of *VAMP1* transcript.

### Linkage Disequilibrium (LD) of the *VAMP1* region

HaploView 3.1 was used to calculate the extent of LD between the 15 variants located on chromosome 12 between positions 6,441,667 and 6,450,104 (*VAMP1*) with a minor allele frequency >1% in the European (CEU) population published by the HapMap project (www.hapmap.org). An r^2^ cut-off of 0.8 was used to group variants into LD blocks. Five LD blocks were identified. One variant from each block was chosen for genotyping in this study.

### Lenti viral preparation

Five shRNA MISSION RNA interference vectors targeting non- and coding *VAMP1* regions were obtained through a partnership agreement between Sigma and the Mayo Clinic RNA Interference Shared Resource. The Virapower lentiviral expression kit (Invitrogen) was used to produce lentiviral particles in the packaging cell line 293FT according to the manufacturer’s protocol. Viral particles present in the cell culture medium were concentrated by centrifugation through a 20% sucrose cushion for 2 h at 20,000 rpm. The pelleted viral particles were resuspended in PBS and filtered through 0.22 μm centrifugal filters (Millipore). Titers of the viral preparations were measured using the Lenti-X™ qRT-PCR Titration Kit from Clontech. Viral preparations of the five constructs with similar titers (~10^10^ copies/ml) were tested in primary neuronal cultures and clone NM_009496.2-462s1c1 (targeting the coding region of *VAMP1*) gave the best knockdown efficiency and was used in the experiments reported in this paper.

### Primary neuronal culture and viral infection

The cortex from newborn mouse pups were dissected in HIBERNATE™ A media without calcium (BrainBits), and incubated in 1 mg/ml papain (Fisher Scientific) at 30°C for 30 min. Tissue was dissociated by triturating with a series of Pasteur pipettes of decreasing diameter. Following centrifugation to collect the cell pellet, the cells were resuspended in Neurobasal A (Invitrogen) supplemented with B27, GMAX, and bFGF (Invitrogen). Neurons were seeded at a density of 1 × 10^6^ cells/well in polylysine coated 6-well plates. To knockdown *VAMP1* expression after 4 days in culture, 1 ml of medium was removed and 50 μl of viral particles in PBS were added. The following day the virus-containing medium was replaced with 2 ml of Neurobasal A medium.

### Western blot of VAMP1

Primary neuronal cells and mouse brains were extracted with RIPA buffer (Sigma). Insoluble material was pelleted by centrifugation for 5 min at 10,000 g, and the protein concentration in the supernatants was determined by BCA assay (Thermo Scientific). Twelve μg of protein from primary neurons and 30 μg from brain extracts were loaded on 4-20% gradient Tris-glycine Novex gels (Life Technologies). The transfer of proteins to nitrocellulose membranes was carried out at 30 volts for 2 h. The membranes were blocked for 1 h with 5% milk in PBS, incubated overnight with a rabbit VAMP1 antibody (Synaptic Systems) at 1-100 dilution, and finally with an anti-rabbit HRP antibody for 1 h at room temperature at 1-2000 dilution. The blots were developed with SuperSignal West Femto reagent (Pierce), imaged with the Fujifilm Luminescent Image Analyzer LAS4000 System, and the bands were quantitated using ImageQuant software.

### Extraction of soluble Aβ from brain homogenates

Soluble proteins were extracted from mouse brains following a diethlamine (DEA) extraction. Briefly, brains were homogenized in 0.2% DEA (in 50 mM NaCl) at a concentration of 100 mg tissue/ml on ice. The homogeates were centrifuged at 100,000 g for 1 hr at 4°C. Supernatants were removed and neutralized by adding 1/10th volume 0.5 M Tris HCl pH 6.8 and vortexed. The soluble Aβ peptides were immediately quantified by ELISA.

### Aβ ELISA

Levels of Aβ1-40 and Aβ1-42 peptides were quantified using “INNO-BIA plasma Aβ forms” (Innogenetics NV, Ghent, Belgium), a multiplex microsphere-based xMAP technology research use-only reagent kit, on a Luminex 200, according to the manufacturer’s instructions. The INNO-BIA kit uses monoclonal antibodies covalently coupled to spectrally specific fluorescent beads to detect Aβn-42 (mAβN; VFFAEDVG and mAβ42; VGGVVIA) and Aβn-40 (mAβN; VFFAEDVG and mAβ40; VGGVV). These recognition sites are equivalent in murine and human APP. Detection of murine Aβ was comparable to that using the well established Aβ antibodies, BNT77/BAN50 (Aβx-40) and BNT77/BC05 (Aβx-42) that have been used previously to detect murine Aβ [21].

### Preparation of *VAMP1* constructs for luciferase assay

AttB-tagged PCR products containing *VAMP1* sequence were cloned into a pGL3 vector containing an SV40 promoter and Luciferase gene (Promega) using the Gateway cloning system (Invitrogen). Three sets of AttB-flanked primers specific to *VAMP1* sequence 25 bp either side of the three VAMP1 polymorphisms were used to amplify genomic DNA extracted from individuals known to be homozygous for the major or minor alleles. PCR reactions were performed in a reaction mix containing 1×PCR buffer containing 1.5 mM MgCl2 (QIAGEN), 1 mM dNTPs (Promega), 0.2 μM each primer, 2.5U HotStar Taq DNA polymerase and 20 ng genomic DNA to a final volume of 25 μl. Amplification conditions were as follows; 5 minutes at 95°C, followed by 35 cycles of 30 seconds at 95°C, 1 minute at 54°C (rs7390 and rs2072376) or 58°C (rs12964), 1 minute at 72°C and finally an extension step of 10 minutes at 72°C. The resultant amplicons (major and minor allele) were extracted from an ethidium bromide-stained agarose gel using a QIAquick Spin kit (QIAGEN) and verified by sequencing (Mayo Clinic, Rochester). The attB-flanked fragments were integrated via bacterial recombination into a kanamycin-resistant pDONR 221 vector using the BP Clonase II system (Invitrogen) to produce an entry clone. Entry clones were transformed into Library efficiency DH5α chemically competent E.coli (Invitrogen) and grown on LB agar containing 50 μg/ml kanamycin overnight at 37°C. Single colonies were picked for inoculation in liquid LB broth containing 50 μg/ml kanamycin and incubated overnight in a shaking incubator at 37°C. Plasmids were extracted from the bacterial cells using a QIAprep spin kit (QIAGEN). Final expression clones were constructed by recombination of the entry clones with ampicillin-resistant pGL3 promoter vector using the LR Clonase II system (Invitrogen). Expression clones were transformed into DH5α E.Coli and grown on LB agar containing 100 μg/ml ampicillin and single colonies were inoculated in LB broth containing 100 μg/ml ampicillin. Plasmids were extracted using endotoxin-free Zyppy Plasmid miniprep kit (Zymo research) and verified by sequencing. Four expression clones were made in total for each VAMP1 SNP; two constructs for each of the major or minor sequence positioned either 5′ to the SV40 promoter and luciferase reporter gene or 3′ to the luciferase gene.

### Cell culture and transfection of HepG2 cells

Human HepG2 hepatocellular carcinoma immortalized cell lines were supplied by ATCC. Cells were cultured in Eagle Minimum Essential Medium (EMEM) supplemented with 10% fetal bovine serum, 2 mM L-Glutamine, 1X non-essential amino acids, 1000 U/ml Penicillin-Streptomycin (Sigma), 2.5 μg/ml Fungizone (Invitrogen). All cultures were incubated at 37°C in 5% CO_2_. 3×10^5^ cells were plated in 12-well culture plates 24 hours before transfection. Cells were co-transfected in triplicate with the *VAMP1* luciferase expression clones (constructs for each SNP were tested independently) and a pRL vector (Promega) containing Renilla Luciferase reporter gene. Control wells included co-transfection of pGL3C (containing an SV40 promoter and SV40 enhancer) with pRL. On the day of transfection, cells were washed twice with PBS and media was replaced with 400 μl serum-free EMEM containing 200 ng expression clone or control vector, 10 ng pRL and transfection reagent Tfx-20 (Promega) at a charge ratio of 3:1 (Tfx:DNA) per well. Transfection mix was pre-incubated for 15 minutes at room temperature. One hour after transfection, 800 μl complete EMEM was added to each well.

### Dual luciferase assay

48 hours after transfection, cells were washed twice with PBS and harvested with 200 μl of 1× Lysis buffer (Promega) for 20 minutes on a rocking platform. 5 μl lysate was plated in a white 96-well assay plate. Firefly and Renilla luciferase signal were measured on a Veritas microplate luminometer (Turner Biosystems) using the dual luciferase reporter assay system (Promega). The ratio of Firefly to Renilla luciferase signal was used to normalize firefly activity for intra-experimental transfection efficiency. Unpaired t-tests comparing mean relative firefly signal for our expression clones were performed.

### Statistical analyses

As the ΔC_T_ trait was found to follow a Gaussian distribution (Kolmogorov-Smirnov p = 0.19), parametric analyses were used. Linear regression of *VAMP1* mRNA levels (ΔC_T_) with genotype and logistic regression of genotype with disease status were performed assuming dominant, additive and recessive models and adjusting for possession of the *APOE* ε4 allele, sex and age. Meta-analyses (random effects DerSimonian-Laird method) of the odds ratios and heterogeneity (Tau-square) for each case-control subseries were performed for rs2072376 (recessive model), rs74056956 and rs71584834 (dominant model). All statistics were performed using SPSS v22 software.

## Additional files

Additional file 1:
**Table S1.** Association of *VAMP1* genotypes with *VAMP1* mRNA expression in Epstein-Barr virus-transformed lymphoblastoid cell lines as published previously by Dixon et al. The polymorphism ID (rs), Chromosomal position (base pairs), allele tested, linkage disequilibrium (LD) block and association levels (heritability; H2, effect, logarithm of odds; LOD and p-value) for three *VAMP1* mRNA transcripts are shown for polymorphisms lying within the *VAMP1* region. Average VAMP1; average coefficient across all three transcripts associated with each variant. **Table S2.** *VAMP1* genotype counts and minor allele frequencies (MAF) in A) postmortem autopsy-confirmed samples and B) case-control series. **Table S3.** Association of *VAMP1* genotypes and *VAMP1* mRNA transcript levels in postmortem cerebellum samples. The number of samples and mean deltaCt values for each group (according to the number of minor alleles assuming dominant, additive or recessive models) are shown. Linear regression statistics (β co-efficient, +/- standard error, T statistic and p-value) adjusted for age, sex and *APOE* ε4 status are shown for each model. The best model (lowest p-value) for each variant is highlighted in yellow. **Table S4.** Association of *VAMP1* genotypes with LOAD risk. Linear regression statistics (β co-efficient, 95% confidence intervals and p-value) adjusted for age, sex and *APOE* ε4 status are shown for each variant assuming a dominant, additive or recessive model. The results are shown for all combined and each individual series. **Table S5.** Genotype counts and minor allele frequencies (MAF) for rare *VAMP1* variants in our case-control series. Additional file 2: Figure S1. Linkage Disequilibrium (LD) between variants with a minor allele frequency >5% in the *VAMP1* and 3′untranslated region (UTR). Genotype data from the Caucasian European (CEU) population published at www.hapmap.org. The location of the polymorphisms (marked by the genotyped alleles) genotyped by HapMap is provided in the top box. The box also includes the exonic (yellow box) and UTR (grey box) regions for the 3 common *VAMP1* transcripts. Below, the pairwise r2 values are given within each box (where r2 = 100, no number is shown). The r2 cutoff for grouping polymorphisms within the same LD block was r2 ≥ 80 (indicated by black boxes). The LD block assigned to each variant is shown in the white circles. One variant from each LD block (*) was chosen as a tagging variant for that block and genotyped in our study. Below, the LD for the 5 variants genotyped in this study is shown based on the genotypes in our case-control series. Each polymorphsim is labelled with the rs number, alleles genotyped (MajorMinor) and chromosomal position.

## Abbreviations

ARUK: Alzheimer’s Research United Kingdom
APP: Amyloid precursor protein
APP: Amyloid precursor protein intracellular domain
LD: Linkage disequilibrium
MAF: Minor allele frequency
SNARE: SNAP (Soluble N-ethylmaleimide-sensitive factor Attachment Protein) REceptors
VAMP1: Vesicle-associated membrane protein 1
Aβ: β-amyloid
