# Erratum to: Genetically-controlled Vesicle-Associated Membrane Protein 1 expression may contribute to Alzheimer’s pathophysiology and susceptibility

**DOI:** 10.1186/s13024-015-0047-2

**Published:** 2015-09-23

**Authors:** Daniel Sevlever, Fanggeng Zou, Li Ma, Sebastian Carrasquillo, Michael G. Crump, Oliver J. Culley, Talisha A. Hunter, Gina D. Bisceglio, Linda Younkin, Mariet Allen, Minerva M. Carrasquillo, Sigrid B. Sando, Jan O. Aasly, Dennis W. Dickson, Neill R. Graff-Radford, Ronald C. Petersen, Ferenc Deák, Kevin Morgan for ARUK consortium, Olivia Belbin

**Affiliations:** Department of Neuroscience, Mayo Clinic College of Medicine, Jacksonville, Fl 32224 USA; Human Genetics Division, Cincinnati Children’s Hospital Medical Center, Cincinnati, OH 45229 USA; Department of Neurology, St. Olav’s Hospital, Edvard Griegs Gate 8, 7006 Trondheim, Norway; Department of Neuroscience, Norwegian University of Science and Technology, NTNU, 7491 Trondheim, Norway; Department of Neurology, Mayo Clinic College of Medicine, Jacksonville, Fl 32224 USA; Department of Neurology and the Mayo Alzheimer Disease Research Center, Mayo Clinic College of Medicine, Rochester, MN USA; Reynolds Oklahoma Center on Aging, Department of Geriatric Medicine, University of Oklahoma HSC, Oklahoma City, OK 73104 USA; School of Molecular Medical Sciences, Institute of Genetics, Queen’s Medical Centre, University of Nottingham, Nottingham, UK; Memory Disorders Unit, Institute of Biomedical Investigation Sant Pau (IIB Sant Pau), Autonomous University of Barcelona (UAB), Barcelona, Spain

## Erratum

Following publication of this work [1], we noticed that we inadvertently failed to include Dr Ferenc Deák in the author list. The author list has now been corrected and the amended authors’ contributions section has been modified accordingly below.
