# Supplementary figures and images for: Genetically-controlled Vesicle-Associated Membrane Protein 1 expression may contribute to Alzheimer’s pathophysiology and susceptibility
Source: Mol Neurodegener. 2015 Apr 9;10:18. doi: 10.1186/s13024-015-0015-x (PMC4426163; doi:10.1186/s13024-015-0015-x)

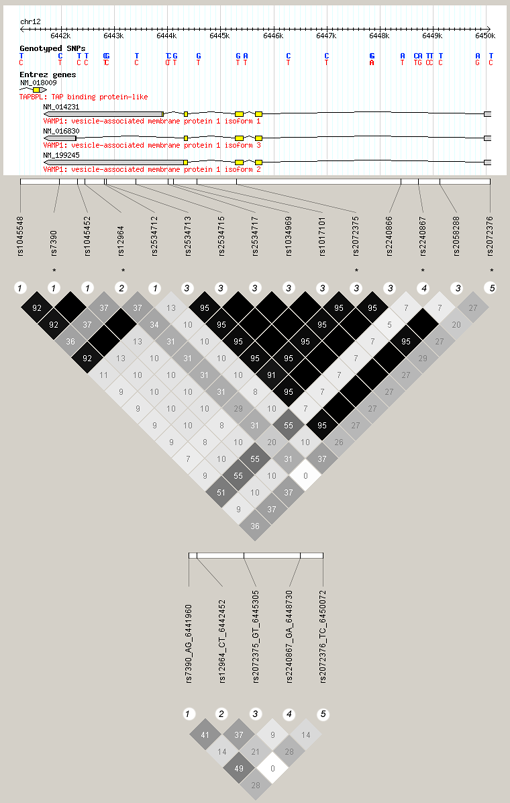

Supplement: Additional file 2: Figure S1. — Linkage Disequilibrium (LD) between variants with a minor allele frequency >5% in the VAMP1 and 3′untranslated region (UTR). Genotype data from the Caucasian European (CEU) population published at www.hapmap.org. The location of the polymorphisms (marked by the genotyped alleles) genotyped by HapMap is provided in the top box. The box also includes the exonic (yellow box) and UTR (grey box) regions for the 3 common VAMP1 transcripts. Below, the pairwise r2 values are given within each box (where r2 = 100, no number is shown). The r2 cutoff for grouping polymorphisms within the same LD block was r2 ≥ 80 (indicated by black boxes). The LD block assigned to each variant is shown in the white circles. One variant from each LD block (*) was chosen as a tagging variant for that block and genotyped in our study. Below, the LD for the 5 variants genotyped in this study is shown based on the genotypes in our case-control series. Each polymorphsim is labelled with the rs number, alleles genotyped (MajorMinor) and chromosomal position. [file 13024_2015_15_MOESM2_ESM.tiff]
